# Peptides of the Constant Region of Antibodies Display Fungicidal Activity

**DOI:** 10.1371/journal.pone.0034105

**Published:** 2012-03-21

**Authors:** Luciano Polonelli, Tecla Ciociola, Walter Magliani, Pier Paolo Zanello, Tiziana D'Adda, Serena Galati, Flavia De Bernardis, Silvia Arancia, Elena Gabrielli, Eva Pericolini, Anna Vecchiarelli, Denise C. Arruda, Marcia R. Pinto, Luiz R. Travassos, Thelma A. Pertinhez, Alberto Spisni, Stefania Conti

**Affiliations:** 1 Sezione di Microbiologia, Dipartimento di Patologia e Medicina di Laboratorio, Università degli Studi di Parma, Parma, Italy; 2 Sezione di Anatomia ed Istologia Patologica, Dipartimento di Patologia e Medicina di Laboratorio, Università degli Studi di Parma, Parma, Italy; 3 Dipartimento di Genetica, Biologia dei Microrganismi, Antropologia, Evoluzione, Università degli Studi di Parma, Parma, Italy; 4 Dipartimento di Malattie Infettive, Parassitarie ed Immunomediate, Istituto Superiore di Sanità, Rome, Italy; 5 Sezione di Microbiologia, Dipartimento di Medicina Sperimentale e Scienze Biochimiche, Università degli Studi di Perugia, Perugia, Italy; 6 Unidade de Oncologia Experimental e Disciplina de Biologia Celular, Departamento de Microbiologia, Imunologia e Parasitologia, Universidade Federal de São Paulo, São Paulo, Brazil; 7 Departamento de Microbiologia, Universidade de São Paulo, Brazil; 8 Dipartimento di Medicina Sperimentale, Università degli Studi di Parma, Parma, Italy; Federal University of São Paulo, Brazil

## Abstract

Synthetic peptides with sequences identical to fragments of the constant region of different classes (IgG, IgM, IgA) of antibodies (Fc-peptides) exerted a fungicidal activity *in vitro* against pathogenic yeasts, such as *Candida albicans*, *Candida glabrata*, *Cryptococcus neoformans*, and *Malassezia furfur*, including caspofungin and triazole resistant strains. Alanine-substituted derivatives of fungicidal Fc-peptides, tested to evaluate the critical role of each residue, displayed unaltered, increased or decreased candidacidal activity *in vitro*. An Fc-peptide, included in all human IgGs, displayed a therapeutic effect against experimental mucosal and systemic candidiasis in mouse models. It is intriguing to hypothesize that some Fc-peptides may influence the antifungal immune response and constitute the basis for devising new antifungal agents.

## Introduction

In their basic monomeric structure, antibodies (Abs) or immunoglobulins (Igs) comprise two identical heavy (H) and light (L) chains linked by disulfide bonds [1]. H and L chains contain a variable region with three hypervariable domains (H1, H2, H3 and L1, L2, L3) defined complementarity determining regions (CDRs). CDRs form the two antigen (Ag) binding sites of an Ab and define its specificity while the constant part (Fc) triggers different effector mechanisms through interaction with specific Fc receptors on immune cells, and other immune molecules, such as complement proteins. Five classes of Igs can be distinguished according to the relative diversity of their H chains Fc (IgG, IgM, IgA, IgE and IgD).

In previous studies we demonstrated that the CDRs, or related peptidic fragments, from a recombinant single chain antiidiotypic Ab (KT-scFv), may exert a fungicidal effect *in vitro* against *Candida albicans* [2]. KT-scFv represented the internal image of a *Pichia anomala* killer toxin (KT) characterized by the wide spectrum of antifungal activity against yeasts and molds expressing specific cell wall receptors, mainly constituted by β-glucans. A decapeptide, designated as killer peptide (KP), derived by alanine substitution from a fragment comprising part of KT-scFv CDR L1 (P6), proved to be active, *in vitro*, against different pathogenic fungi and to exert effective therapeutic activity in experimental models of vaginal and systemic candidiasis, disseminated cryptococcosis and paracoccidioidomycosis [3]. KP, which proved to be devoid of cytotoxicity on human PBMCs and mammalian cells, is characterized by a peculiar self-aggregation-releasing property, catalyzed by β 1,3-glucan, which might be at the basis of its unexpected pharmacokinetic characteristics [4].

According to the frequent inclusion of P6 within the sequences of the V regions of many Abs, as can be seen in available databases, it was hypothesized that isolated CDRs may display antifungal activities irrespective of Ab specificity for a given Ag [5]. CDR-based synthetic peptides of murine and human monoclonal (m)Abs, characterized by unrelated specificities, showed fungicidal activities *in vitro* and/or *in vivo* against various fungi, conceivably involving different mechanisms of action. Engineered peptides, obtained by alanine substitutions of CDR sequences, showed increased, unaltered or decreased antifungal activities [5]. The finding suggests that Abs may be sources of an unlimited number of sequences potentially active against pathogenic fungi [3].

Although of interest, the potential antifungal activity of Ab CDRs may be considered of relative importance from an immunological point of view, since it is unlikely that a significant amount of the specific fragments should be released *in vivo*. Here we report on the selection and synthesis of peptides encompassing sequences of the Fc (Fc-peptides) from the major classes of Igs (IgG, IgM, IgA), and the evaluation of their antifungal activity. Fc-peptides with fungicidal activity, that might be released *in vivo* by proteolysis of Igs, may represent a link between adaptive and innate immunity playing a role against fungal infections. Such peptides, moreover, could constitute novel lead molecules for devising new antifungal agents.

## Materials and Methods

### Ethics statement

Procedures involving animals and their care were conducted in conformity with national and international laws and policies. The study has been approved by the Committees on the Ethics of Animal Experiments of the University of Perugia (Permit Number: 41-2005B and 34/2003-A) and Istituto Superiore di Sanità, Rome (Permit Number: DM 227/2009-B dated 12/21/2009).

### Selection and synthesis of Fc-peptides and their derivatives

**Figure 1:**
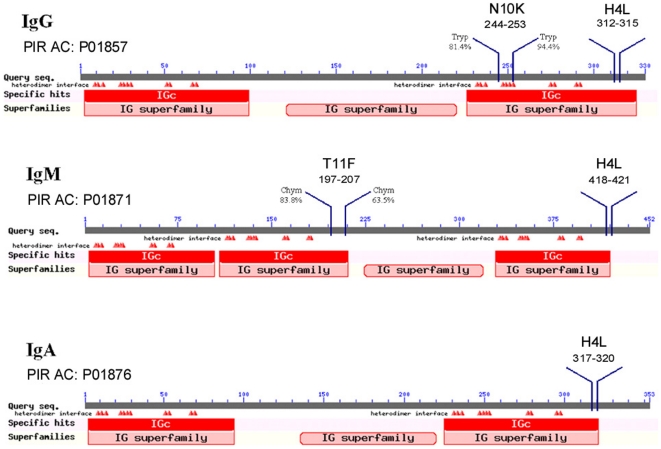
Localization of the selected peptides within the constant region of different antibody isotypes.

Abs sequences were found in the Protein Information Resource (PIR) database (http://pir.georgetown.edu/). The selection of Fc-peptides was made by using the following analysis tools: BLAST (http://blast.ncbi.nlm.nih.gov/Blast.cgi); ClustalW (http://www.ebi.ac.uk/Tools/msa/clustalw2/); ExPASy Proteomics Tools (http://expasy.org/tools/); The Sequence Manipulation Suite 2 (http://www.bioinformatics.org/sms2/index.html). The definition of Fc-peptides of interest within each Ig class (IgG, IgM and IgA) was made according to different criteria: peptide length (maximum 12 amino acids), potential cleavage sites by trypsin and/or chymotrypsin-high specificity proteases, cleavage probability of the individual sites, conserved amino acids in human Ig and in different organisms, isoelectric point, and alternation of hydrophobic/hydrophilic residues in the sequence. Selected Fc-peptides (Table 1, Fig. 1) were synthesized, to be used for the studies of *in vitro* fungicidal and *in vivo* therapeutic antifungal activity, using Fmoc solid-phase synthesis chemistry on a Syro II Peptide Synthesizer (MultiSynTech, Germany) at CRIBI (Biotechnology Centre, University of Padova, Italy).

**Table 1:** Characteristics of peptides selected from the constant region of antibodies.

Peptide	Amino acid sequence	Ig class	Proteases involved in cleavage	%^a^	Hydrophobicity^b^	pI	M.M. (Da)	Net charge
H4L	HEAL	IgG, IgM, IgA^c^	-	-	+-00	5.12	468.49	−
N10K	NQVSLTCLVK	IgG1^c^	Tryp+Tryp	81,4	**0*0**00+	8.23	1104.33	+
T11F	TCRVDHRGLTF	IgM^d^	Chym+Chym	63,5	**+0-++00*0	8.27	1304.51	+

a cleavage probability;

b 0: hydrophobic, *: polar, +, and −: positively charged and negatively charged residues;

c conserved amino acids in human Ig and different organisms;

d conserved amino acids only in human Ig;

pI, isoelectric point; M.M., molecular mass (dalton); Tryp, trypsin; Chym, chymotrypsin.

An irrelevant synthetic decapeptide (MSTAVSKCAT), previously proven to be devoid of fungicidal activity [2], was synthesized to be used in some experiments as a negative control.

Selected Fc-peptides proven to exert fungicidal activity *in vitro* were analysed by alanine-scanning. Alanine-substituted derivatives (asds), defined according to the position held by the alanine-substituted amino acid, were tested in selected experiments to critically establish the functional relevance of each residue.

### Fungal strains

The following fungal strains were employed for the evaluation of the fungicidal activity of selected Fc-peptides: *C. albicans* SC5314 a well known reference laboratory strain; *C. albicans* CA-6, a highly virulent strain whose origin and characteristics have previously been described [6] (from the fungal collection of the Microbiology Section, Department of Experimental Medicine and Biochemical Sciences, University of Perugia, Italy); *C. albicans* SA40 and AIDS68, fluconazole susceptible and resistant strains, respectively, originally isolated from a human vaginal infection (from the fungal collection of the Department of Infectious, Parasitic and Immuno-mediated Diseases, Istituto Superiore di Sanità, Rome, Italy); *C. albicans* UM4, caspofungin resistant, and *Candida glabrata* OMNI32, fluconazole, itraconazole, and voriconazole resistant (from the fungal collection of the Department of Public Health, University of Milan, Italy); *C. neoformans* var. *neoformans* 6995, a capsulated serotype A strain (University of Perugia), and *Malassezia furfur* 101 (a clinical isolate from the collection of the University of Parma). *C. albicans* 64548 from the American Type Culture Collection was used in transmission electron and confocal microscopy studies.

### Evaluation of the *in vitro* fungicidal activity of selected Fc-peptides

Selected Fc-peptides, or their asds, were preliminarily tested at the concentration of 100 µg/ml. Peptides exhibiting fungicidal activity at this concentration were further tested at scalar dilutions to determine the half maximal effective concentration (EC_50_) values. Conventional colony forming unit (CFU) assays were performed as previously described [2]. Briefly, yeast cells from a fresh (20 h at 30°C) actively growing colony on Sabouraud Dextrose agar (SDA) (*C. albicans*, *C. glabrata*, *C. neoformans*) or on SDA supplemented with 1% Tween 20 (*M. furfur*) were suspended in 199 medium. Initial germination was induced in *C. albicans* cells by incubation for 1 h at 37°C with shaking. Suspensions were properly diluted to obtain approximately 500 fungal cells in 100 µl of distilled water in the presence or absence (control growth) of the synthetic Fc-peptide to be tested. Cell suspensions were incubated at 37°C for 6 h, then plated on SDA or SDA-Tween. CFU were enumerated after 48–72 h of incubation at 30°C, and peptide fungicidal activity was determined as percentage of CFU inhibition, according to the formula 100-(CFU Fc-peptide treated/CFU control growth)×100. Each assay was carried out in triplicate for statistical purposes. In preliminary experiments, the decapeptide MSTAVSKCAT was used as additional control.

EC_50_ was calculated by nonlinear regression analysis using Graph Pad Prism 4.01 software, San Diego, CA, USA.

### Evaluation of the hemolytic, cytotoxic, and genotoxic effects of selected peptides (H4L, N10K and its asd N1A, T11F and its asd D5A)

Selected FC-peptides and asds were tested for their hemolytic activities against human red blood cells (group 0 Rh+) (hRBC). Freshly collected hRBC with EDTA were rinsed 3 times with phosphate buffered saline (PBS) by centrifugation for 10 min at 1300 *g* and resuspended in PBS. Serial dilutions of Fc-peptides in PBS were then prepared and added to 150 µl of the stock hRBC solution to a final concentration of 500, 250, 100 and 50 µg/ml in the volume of 300 µl (final erythrocyte concentration, 2.5% v/v). The resulting suspensions were incubated with shaking for 30 min and 2 h at 37°C. 100 µl of the samples were then centrifuged at 800 *g* for 10 min. Release of hemoglobin was monitored by measuring the absorbance of the supernatant at 540 nm. Controls for zero hemolysis (blank) and 100% hemolysis consisted of hRBC suspended in PBS and Triton 1%, respectively.

Cytotoxicity against LLC-MK2 monkey kidney epithelial cells was determined by MTT assay, based on the ability of metabolically active cells to convert the yellow water-soluble tetrazolium salt into formazan crystals. LLC-MK2 cells cultured in MEM containing 10% fetal bovine serum (FBS) were seeded in 96-well plates (1×10^5^ cells/ml, 100 µl/well) and incubated for 24 h at 37°C in 5% CO_2_ atmosphere. The cells were then treated with the selected peptides in medium containing 2% FBS (final concentrations 50, 100, 250 e 500 µg/ml) for 24 h. Cells in medium without peptides served as control. After this period, cells were incubated with MTT (5 mg/ml, 10 µl/well) in serum-free medium for 2 h at 37°C, the medium was removed and the crystal formazan dye was solubilized by adding 100 µl isopropanol (with 5% HCl 1 M). Absorbance was measured at 540 nm.

To detect if selected Fc-peptides might induce DNA damage, Alkaline Comet assay was performed on PBMCs. PBMCs were obtained from healthy donor whole blood by Lymphoprep™ density gradient and, after two washes in HBSS, cultured at a concentration of 1×10^6^ cells/ml in RPMI 1640 containing 10% FBS, 1% phytohemoagglutinin, 1 mM L-glutamine, 100 IU penicillin, and 100 µg/ml streptomycin. PBMCs were treated for 2 h with different concentrations of the selected peptides (5 µM, 10 µM, 20 µM) at 37°C in 5% CO_2_ atmosphere. The Comet assay was performed as described by Singh *et al.* [7], with minor modifications. Cell lysis was carried out at 4°C overnight by exposing the cells to a buffer containing 2.5 M NaCl, 10 mM Na_2_EDTA, 10 mM Tris-HCl, 1% Triton X-100 and 10% DMSO, pH 10. DNA unwinding was achieved over 20 min in an electrophoretic alkaline buffer (1 mM Na_2_EDTA, 300 mM NaOH, 0°C, pH>13), electrophoresis was then carried out for 20 min, (0.78 V/cm, 300 mA) at 0°C in the same buffer, followed by neutralisation in 0.4 M Tris-HCl, pH 7.5. Immediately before microscopic examination, the slides were stained with 0.75 µl ethidium bromide (10 µg/ml). The slides were examined with a fluorescent microscope (Leica DMLS), equipped with a BP 515–560 nm excitation filter and an LP 580 nm barrier filter, and data were collected using an automatic image analysis system (Comet Assay III, Perceptive Instruments Ltd). Fifty randomly-selected cells per slide (two slides per sample) were analyzed. The samples were coded and evaluated blind. DNA migration was evaluated by percentage of DNA in comet tail (Tail Intensity, expressed as mean of the median values).

### Evaluation of the therapeutic activity of a selected Fc-peptide

#### Experimental systemic infection

Female Balb/c mice (Harlan Nossan Laboratories), 8–10 weeks old, were housed at the Animal Facilities of the University of Perugia, Perugia, Italy. Groups of 8 animals were infected intravenously with 5×10^5^ or 7.5×10^5^ *C. albicans* CA-6 cells suspended in 0.5 ml saline solution, for evaluation of fungal clearance or mice survival, respectively. Infected animals were given 200 µg of N10K peptide intraperitoneally 1, 24, and 48 h after infection for a total dose of 600 µg/mouse. Infected animals treated with the same amount of the negative control peptide or untreated (i.e. injected with the peptide solvent) served as controls. Quantification of fungal renal burden at 7 and 12 days after infection was assessed by plating serial dilutions of kidney homogenates onto SDA plates. Animal survival was monitored up to 60 days after infection.

#### Experimental vaginal infection

Female Balb/c mice (18–21 g) (Charles River) were housed at the Animal Facilities of the Istituto Superiore di Sanità, Rome, Italy. Groups of 5 animals were injected subcutaneously with 0.02 mg of estradiol benzoate (Amsa Farmaceutici srl, Rome, Italy) in 100 µl of saline solution, 48 h before inoculation with *Candida* and weekly thereafter. The animals were inoculated intravaginally with 20 µl of saline solution containing 10^6^ cells of *C. albicans* SA40 or AIDS68 strains. The inoculum was dispensed into the vaginal cavity through a syringe equipped with a multipurpose calibrated tip (Combitip; PBI, Milan, Italy). The yeast cells were previously grown in YPD broth (1% yeast extract, 2% peptone, 2% dextrose) at 28°C on a rotatory shaker (200 rpm), harvested by centrifugation (1500 *g*), washed, counted in a hemocytometer, and suspended to the required number in saline solution. N10K was administered intravaginally three times at 1, 24, and 48 h after intravaginal *C. albicans* challenge (20 µg per dose in 20 µl of distilled water) for a total of 60 µg/mouse. Infected animals treated with the same amount of the negative control peptide or untreated served as controls. As a further control, in selected experiments mice received 100 µg of fluconazole (Pfizer) in PBS (20 µl) at 1, 24, and 48 h after the yeast challenge. The number of cells in the vaginal fluid was counted by culturing 100 µl of vaginal samples, taken by washing the vaginal cavity by gentle aspiration of 100 µl of sterile saline solution, repeated four times, at 1∶10 serial dilutions on SDA containing chloramphenicol (50 µg/ml). CFUs were enumerated after incubation at 28°C for 48 h. The outcome of treatment was assessed in terms of acceleration of fungal CFU clearance over a period of 28 days.

### Statistical analysis

Data are reported as the mean ± sd. Statistical significance of survival curve of animals was assessed by the Kaplan-Meier logrank test. Statistical significance of vaginal fungal clearance was assessed by the analysis of variance (ANOVA) and the Bonferroni post hoc test. A value of P<0.05 was considered significant.

### Circular Dichroism (CD) spectroscopy

CD measurements were carried out on a Jasco 715 Spectropolarimeter (JASCO International Co. Ltd.) coupled to a Peltier PTC-348WI system for temperature control. N10K stock solution was 1.8 mM and, to follow the aggregation process, aliquots were diluted to a final concentration of 100 µM. Far-UV spectra were recorded in the spectral range 190–250 nm and averaged over four scans, using a 1 mm path length quartz cell, at 20°C. Following baseline correction, the measured ellipticity, θ (mdegree), was converted to the molar mean residue ellipticity [θ] (deg·cm^2^·dmol^−1^). The spectra of N10K (1 mM) in the presence of *C. albicans* (5×10^7^ cells/ml) were collected at 20°C using a 0.1 mm path length cell.

### Transmission electron microscopy studies

For the electron microscopy studies, aliquots of peptide solution (N10K and negative control peptide) were applied to Formvar-coated grids, and negatively stained with a solution of 2% (w/v) uranyl acetate in water. The grids were washed, air-dried, and then examined in a Philips EM 208S transmission electron microscope (Fei Europe, Eindhoven, The Netherlands) operating at an accelerating voltage of 80 kV.

For transmission electron microscopy studies on yeasts, *C. albicans* ATCC 64548 cells (approximately 10^7^) were incubated in the absence (control) or presence of N10K (150 µg) for 1 h. Negative control peptide served as an additional control. Cells were fixed with 2.5% glutaraldehyde in sodium cacodylate [Na(CH_3_)_2_ AsO_2_ • 3H_2_O] buffer. After washing, 4 times, 15 min each, with 100 mM cacodylate buffer, pH 7.4 at 4°C, the pellet was post-fixed in the same buffer with 1% osmium tetroxide for 2 h. Dehydration was carried out by immersion in ethanol in a 70% to 100% gradient (twice for 10 min at 70%; twice for 10 min at 95% and 4 times for 15 min at 100%). The pellet was then incubated 2 times (15 min each) in 100% propylene oxide at room temperature followed by a mixture of propylene oxide and Spurr resin (v/v) at room temperature for 12 h. Materials were then kept in pure resin for 5 h, at room temperature, and then in a new solution of pure resin at 70°C for 72 h. Semi-thin cuts (300 nm) were stained with 0.25% toluidine blue for observation in an optical microscope (Axiophot Zeiss, with plan achromatic objective), followed by ultra-thin cuts (70 nm) which were contrasted with 0.5% uranyl acetate and 0.5% lead citrate and observed in an EM Jeol 100 CX transmission electron microscope.

### Confocal microscopy studies

*C. albicans* ATCC 64548 cells (2×10^7^) were incubated for 1 h at room temperature with biotinylated N10K, washed 3 times with PBS and fixed with 4% paraformaldehyde for 1 h. The cells were permeabilized in 0.2% Triton X-100 for 1 h and blocked with buffer (150 mM NaCl, 50 mM Tris and 0.25% BSA) for 1 h. Permeabilized cells were then incubated for 1 h in the dark with streptavidin-fluorescein isothiocyanate (Invitrogen, Camarillo, CA) diluted 1∶200 in PBS. Cells were then washed and stained with phalloydin-rhodamine (1∶1000) (Invitrogen) for 1 h, followed by DAPI (1∶3000) (Invitrogen) staining for 1 h. Cells were washed 3 times with PBS and 40 µl of Vectashield® mounting medium (Sigma) was added to the pellet. A sample of 20 µl was mounted on coverslips and observed in a Confocal Leica SP5 microscope, with a 63×1.4 oil objective; the Z series was obtained according with sampling criteria built in the software. DAPI, which stains the nucleus in blue, was examined at 350 nm excitation and 470 nm emission, and the streptavidin-FITC at 494 nm excitation and 520 nm emission. For phalloidin-rhodamine that stains filamentous actin in red, the excitation at 580 nm and emission at 604 nm were used.

## Results

### Properties of selected Fc-peptides

The amino acid sequences and properties of the selected peptides are shown in Table 1. Their localization within the constant region of the different classes of Abs is shown in Figure 1. In particular, H4L peptide was selected because it occurs in the C region of all human and mammalian IgAs, IgGs, and IgMs. N10K, a fragment of human IgG1 conserved in different organisms, and T11F, a fragment of human IgMs, resulting from the digestion by trypsin and chymotrypsin, respectively, were selected according to their high isoelectric point, and the alternation of hydrophobic/hydrophilic residues in the sequence, with regard to our previous experience in the field of antifungal Ab peptides.

### *In vitro* fungicidal activity of selected Fc-peptides

The *in vitro* fungicidal activity of the selected peptides was assessed by CFU assays. N10K and T11F exhibited significant activity against all the investigated fungal strains, while H4L showed a weaker activity (Table 2). The negative control peptide, recognized to be devoid of anti-*Candida* properties [2], proved, in preliminary assays performed at the concentration of 100 µg/ml, to have no fungicidal activity against the tested strains (data not shown). EC_50_ values, determined for the two most active Fc-peptides, ranged between 0.1 and 3.5 µM. In comparison to the parental Fc-peptides N10K and T11F, their alanine substituted derivatives were tested in the same biological assay against *C. albicans* SC5314 in order to study the role of each residue in antifungal activity. Results (Tables 3 and 4, respectively) showed a candidacidal activity significantly increased or decreased, with the only exception of T11F asd G8A whose EC_50_ value was comparable to the one of the parental Fc-peptide.

**Table 2:** *In vitro* fungicidal activity of the selected Fc-peptides against fungal strains.

	Fungicidal activity					
	N10K		T11F		H4L	
Fungal strain	%*	EC_50_ **(95% confidence intervals) [mol/liter]×10^−6^	%	EC_50_ (95% confidence intervals) [mol/liter]×10^−6^	%	EC_50_
*C. albicans* SC5314	100	1.004 (0.947–1.065)	100	1.540 (1.404–1.689)	84.34	n.d.
*C. albicans* CA-6	100	2.939 (2.578–3.351)	100	0.518 (0.506–0.531)	57.04	n.d.
*C. albicans* SA40	100	1.763 (1.670–1.861)	100	0.286 (0.251–0.326)	55.99	n.d.
*C. albicans* AIDS68	100	1.745 (1.396–2.180)	100	0.295 (0.254–0.341)	59.82	n.d.
*C. albicans* UM4	100	2.784 (2.622–2.956)	100	0.667 (0.636–0.700)	64.63	n.d.
*C. glabrata* OMNI32	100	3.584 (3.390–3.788)	100	0.252 (0.245–0.260)	80.49	n.d.
*C. neoformans* 6995	100	2.948 (2.645–3.288)	100	0.811 (0.632–1.041)	85.08	n.d.
*M. furfur* 101	100	2.833 (1.900–4.226)	100	0.115 (0.103–0.129)	92.04	n.d.

* % inhibitory activity at 100 µg/ml in comparison to the control growth (water);

** EC_50_, half maximal effective concentration;

n.d., not determined.

**Table 3:** *In vitro* comparative fungicidal activity of N10K alanine-substituted derivatives against *Candida albicans* SC5314.

		Anti-*Candida* activity		
asd^1^	M.M. (Da)	%*	EC_50_ **(95% confidence intervals) [mol/liter]×10^−6^	EC_50_ *asd*/EC_50_ N10K
N1A	1061.30	100	0.587 (0.586–0.588)§	0.58
Q2A	1047.28	100	2.305 (2.260–2.350)§	2.30
V3A	1076.28	100	5.929 (5.064–6.941)§	5.91
S4A	1088.33	100	2.391 (2.076–2.755)§	2.38
L5A	1062.25	100	0.885 (0.841–0.931)§	0.88
T6A	1074.30	100	4.878 (4.364–5.453)§	4.86
C7A	1072.27	73.41	54.874 (26.243–114.710)§	54.66
L8A	1062.25	100	0.744 (0.732–0.755)§	0.74
V9A	1076.28	100	1.776 (1.742–1.810)§	1.77
K10A	1047.23	0	n.d.	n.d.

1 asd, alanine-substituted derivative; M.M., molecular mass (dalton);

* % inhibitory activity at 100 µg/ml in comparison to the control growth (water);

** EC_50_, half maximal effective concentration;

§ Anti-*Candida* activity significantly different from that of N10K peptide as assessed by *t* test (P<0.001).

n.d., not determined.

**Table 4:** *In vitro* comparative fungicidal activity of T11F alanine-substituted derivatives against *Candida albicans* SC5314.

		Anti-*Candida* activity		
asd^1^	M.M. (Da)	%*	EC_50_ **(95% confidence intervals) [mol/liter]×10^−6^	EC_50_ *asd*/EC_50_ T11F
T1A	1274.45	100	0.738 (0.711–0.766)§	0.48
C2A	1272.41	79.32	32.521 (25.220–41.944)§	21.12
R3A	1219.37	97.61	13.458 (12.146–14.909)§	8.74
V4A	1276.42	100	1.134 (1.083–1.188)§	0.74
D5A	1260.47	100	0.395 (0.389–0.402)§	0.26
H6A	1238.41	100	10.417 (9.948–10.901)§	6.76
R7A	1219.37	100	12.055 (11.498–12.646)§	7.83
G8A	1318.50	100	1.551 (1.465–1.642)	1.01
L9A	1262.39	100	1.753 (1.679–1.830)§	1.14
T10A	1274.45	100	0.850 (0.844–0.854)§	0.55
F11A	1228.38	100	2.519 (2.433–2.607)§	1.64

1 asd, alanine-substituted derivative; M.M., molecular mass (dalton);

* % inhibitory activity at 100 µg/ml in comparison to the control growth (water);

** EC_50_, half maximal effective concentration.

§ Anti-*Candida* activity significantly different from that of T11F peptide as assessed by *t* test (P<0.001, except in the case of L9A, P = 0.0098).

### Hemolytic, cytotoxic, and genotoxic effects of selected Fc-peptides

The Fc-peptides N10K, its most active asd N1A, T11F, its most active asd D5A, and H4L were tested for hemolytic, cytotoxic and genotoxic effects on human red blood cells, mammalian cells and human PBMCs.

All tested peptides showed no hemolytic activity at the concentrations and time investigated, as demonstrated by the statistical analysis of the mean absorbance values of released hemoglobin that did not differ significantly from the ones of the negative control (PBS, 0.03±0.01 at 30 min, 0.05±0.01 at 2 h) in comparison to the positive control (Triton 1%, 1.50±0.01 at 30 min, 1.51±0.11 at 2 h).

None of the investigated peptides was significantly cytotoxic against LLC-MK2 cells as assessed by the MTT assay. At all concentrations tested, mean absorbance values were not different from the ones of the untreated cells with the exception of N10K and N1A asd peptides. In the latter two cases only at the concentration of 500 µg/ml, respectively 450 and 800 times higher than the EC_50_, cell viability was reduced <10% in comparison to untreated control.

None of the tested peptides showed genotoxic activity in the Comet assay performed on PBMCs. No statistically significant increases were observed in % tail DNA in comparison with the negative control value of 0.24±0.05 recorded for untreated PBMC.

### *In vivo* therapeutic activity of N10K

N10K was selected as a proof-of-concept of the therapeutic potential of fungicidal Fc-peptides in consolidated models of murine systemic and mucosal candidiasis.

#### Systemic candidiasis

**Figure 2:**
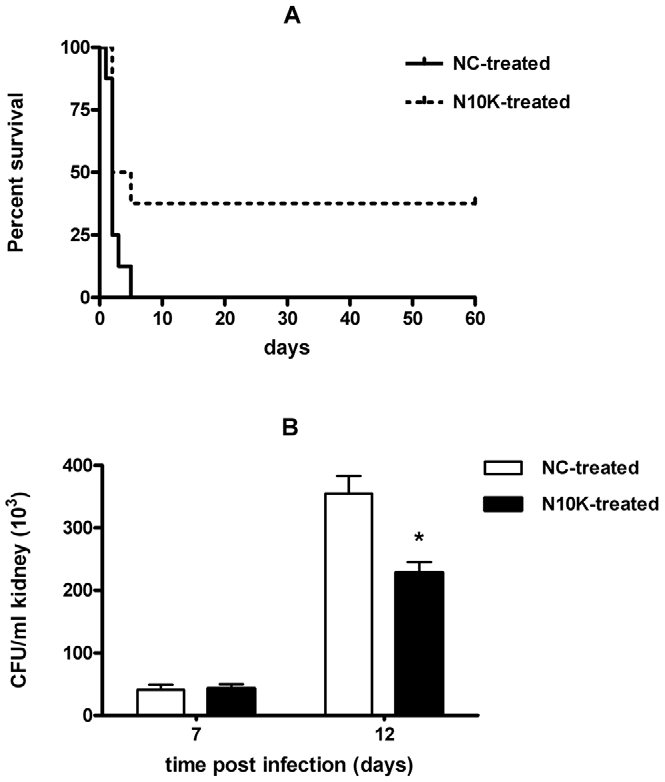
*In vivo* anti-*Candida* activity of N10K in a systemic model of infection. A. Effect of N10K treatment (200 µg, i.e. 180 µM, 1, 24, and 48 h postchallenge) on the survival of mice infected intravenously with 7.5×10^5^ cells of *C. albicans* CA-6. The survival curve of N10K-treated animals was significantly different from that of control mice, treated with an irrelevant peptide (negative control, NC), as assessed by Logrank test (P<0.05). B. Effect of N10K treatment on fungal clearance from kidneys of mice challenged with 5×10^5^ cells of *C. albicans* CA-6. *, P<0.01 N10K-treated *vs* mice treated with the negative control peptide (NC).

Treatment with three doses (200 µg/dose, i.e. 180 µM) of N10K led to a significant increase in survival of mice infected with *C. albicans* CA-6 in comparison to infected mice treated with the same amount of negative control peptide (Figure 2, panel A). N10K treatment significantly reduced fungal burden in the kidneys of infected mice (Figure 2, panel B). Results in animal groups treated with saline were not statistically different from the results obtained in mice treated with the negative control peptide (data not shown).

#### Vaginal candidiasis

**Figure 3:**
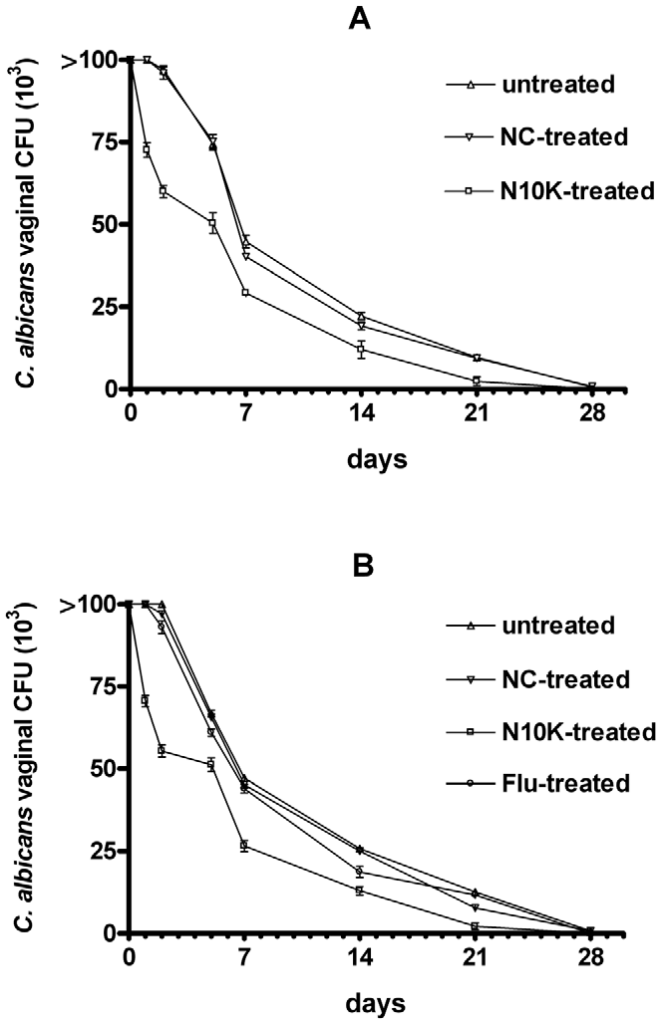
Protection conferred by N10K upon mice intravaginally infected with fluconazole-susceptible strain SA40 (panel A) and fluconazole-resistant strain AIDS68 (panel B) of *C. albicans*. All mice (five per group) were given 10^6^ cells in 20 µl of saline solution on day 0 and were sampled for initial intravaginal colony forming units (CFU). The therapeutics (N10K, 20 µg, i.e. 18 µM; negative control peptide, NC, 20 µg; and fluconazole, Flu,100 µg) were administered 1, 24, and 48 h postchallenge. On days 1, 2, 5, 7, 14, and 21, all the differences in the CFU vaginal counts between N10K-treated and control groups (untreated and NC-treated) were statistically significant (P<0.001). The differences in vaginal CFU counts between N10K-treated and fluconazole-treated AIDS68-infected animals were statistically significant (P<0.001 all days except P<0.01 day 14) on the same days. The statistical significance was assessed by the analysis of variance (ANOVA) and the Bonferroni post hoc test. Data are from one of two experiments performed with similar results.

Administration of three doses (20 µg/dose, i.e. 18 µM) of N10K accelerated the early rate of clearance (1 to 5 days) of the fungus from the vagina of the mice infected with either fluconazole-susceptible SA40 (Figure 3, panel A) or fluconazole-resistant AIDS68 (Figure 3, panel B) *C. albicans* strains. N10K also provided a substantial resolution of the infection (approximately 2×10^3^ CFU/ml of vaginal fluid) within 3 weeks of challenge, when the animals treated with the same amount of the negative control peptide or untreated still had from 7.8×10^3^ to 1.2×10^4^ *Candida* CFU/ml of vaginal fluid. No acceleration of the fungal clearance and no effect on resolution of infection was provided by administration of the negative control peptide. Noteworthy, N10K proved to have a therapeutic effect also in mouse vaginal infection caused by the *C. albicans* strain (AIDS68) which was resistant to treatment with 100 µg of fluconazole (Figure 3, panel B).

### N10K conformational state

**Figure 4:**
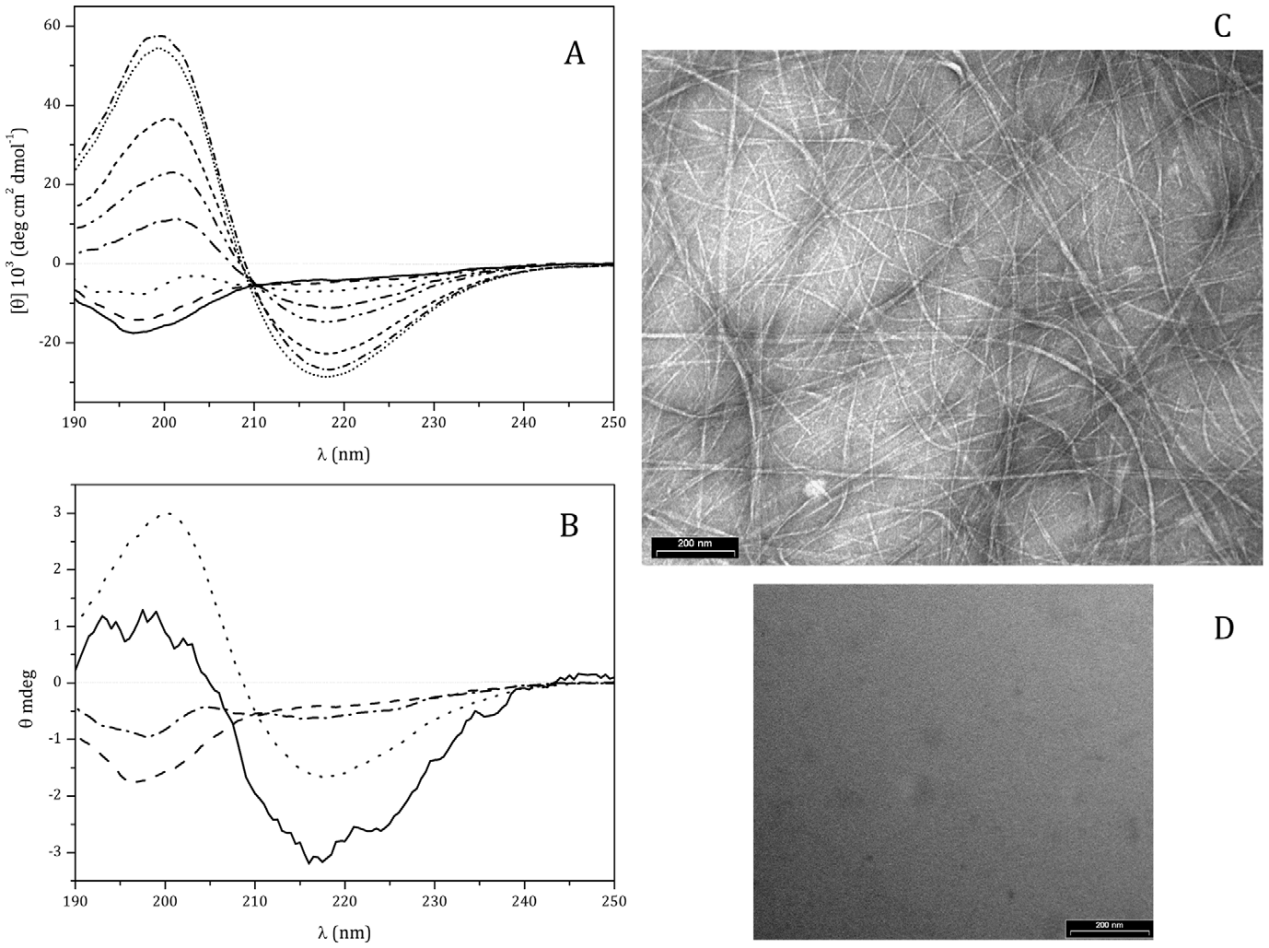
Characterization of N10K conformational transition. A. Far-UV CD spectra of N10K (100 µM) in water, recorded as a function of time: time 0 (^______^); 1 h (— — ); 3 h (- - -); 5 h (— - — -); 7 h (— - - — - -); 20 h (– – –); 6 days (• • •); 20 days (— • — •). B. Interaction of N10K (1 mM) with *C. albicans* (5×10^7^ cell/ml) after 150 min incubation (^______^) using a 0.1 mm path lenght cell, 20°C. Fresh N10K in water (— —); after 150 min incubation (— - — -); after 570 min incubation (- - -). The samples in water were prepared taking aliquots of 100 µM from a 1.8 mM stock water solution and were collected with a 1 mm cell. For the sake of comparison of these CD profiles with the one obtained in the presence of *C. albicans*, the signal intensity of the water CD spectra has been divided by 10 and the ordinate kept in θ (mdeg). C. Electron micrograph of N10K prepared diluting 1∶5 the stock water solution stored at 4°C for 20 days. D. Electron micrograph of negative control peptide prepared in the same conditions.

Studies on the structure-function relationship of N10K peptide carried out by Circular Dichroism spectroscopy revealed its ability to spontaneously aggregate. CD spectra of pure N10K in water (Figure 4, panel A), recorded over time, illustrate the progression of its aggregation, going from a typical random coil conformation to a rich β-sheet structure. In 9 h the peptide reached its final conformation. When N10K was in the presence of *C. albicans*, the CD spectrum recorded at 150 min of incubation was indicative of the onset of β-sheet elements as observed for the pure peptide in water after a much longer incubation time (Figure 4, panel B).

Electron micrographs of the negatively stained N10K demonstrated the presence of a network of fibril-like structures (Figure 4, panel C), while, in the same conditions, negative control peptide showed no aggregation (Figure 4, panel D).

### Visualization of the effects of N10K on *Candida albicans* cells by transmission electron microscopy

**Figure 5:**
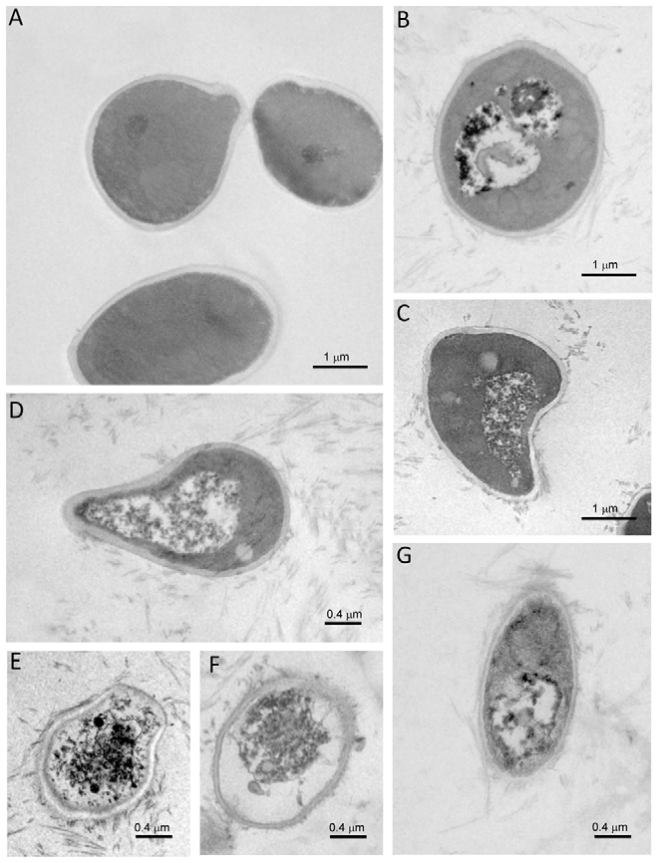
Cytotoxic effects of N10K on *Candida albicans*. Yeast cells were incubated with (panels B, C, D, E, F, and G) or without (panel A) N10K. Original magnification 12000×.

As shown in Figure 5, treatment with N10K caused gross alterations in the morphology of *C. albicans* cells. Aggregates of N10K were observed outside the cells. Masses of disrupted organelles engulfed in single or double compartments were seen (panels B, C, D). Panels E, F, and G show yeast cells with extensive disruption of internal structures. In all cases N10K formed aggregates binding to the cell wall without causing major cell lysis. Treatment with negative control peptide did not affect *Candida* cells (not shown).

### Binding of biotinylated N10K to *Candida albicans* cells

**Figure 6:**
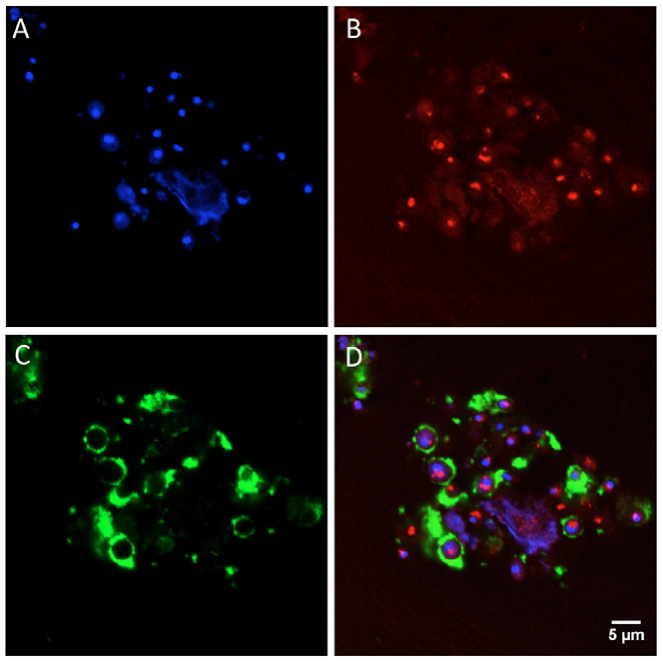
Binding of biotinylated N10K to *Candida albicans* cells. A. Cells stained with DAPI; B. Phalloidin-rodamine stained cells. C. Biotinylated N10K-treated cells stained with streptavidin-fluorescein isothiocyanate. Fluorescence is seen at the cells' periphery, mainly at the cell wall. D. Merge produced by the superposition of the red and green fluorescence outputs of the same cells. The peptide ligand does not colocalize with F-actin.

Biotin-labeled N10K peptide was used in order to evaluate binding to *C. albicans* cells. N10K bound to the surface of *Candida* cells, apparently to a cell wall ligand. Confocal fluorescence microscopy showed that the peptide does not colocalize with phalloidin-rhodamine, a reagent that is quite specific for filamentous actin (Figure 6).

## Discussion

Fungal infections remain a major threat in humans because of the emergence of multidrug resistant strains [8], [9], [10]. Peptide molecules of innate immunity (host defence peptides, HDPs, such as defensins, histatins, etc.) have been recognized to display antifungal activity mainly owing to interactions with cell membranes or inhibition of metabolism [11], [12], [13], [14].

We had shown that, irrespective of the specificity of the native Ab for a given Ag, CDR-related peptides may exert differential *in vitro*, *ex vivo* and/or *in vivo* antimicrobial, antiviral, antitumor and/or immunomodulatory activities, conceivably mediated by different mechanisms of action, reminiscent of molecules of innate immunity [2], [5], [15], [16].

In this work, Fc-peptides putatively arising from the cleavage of the constant region of Abs (IgG, IgM, IgA) by physiological enzymes (trypsins, chimotrypsins) proved to display *in vitro* antifungal activity against fungal pathogens, such as *C. albicans*, *C. glabrata*, *C. neoformans*, and *M. furfur*. N10K and T11F asds, used in order to identify the functional contribution of each residue, showed increased, unaltered or decreased candidacidal activity in comparison to parental peptides. Replacement of cysteine in both N10K and T11F, that hampers disulphide bond formation, determined the most significant reduction of candidacidal activity, followed by substitution of positively charged residues (lysine in N10K, histidine and arginine in T11F). In the case of N10K, the substitution of the unique positively charged residue that makes the peptide highly hydrophobic (with net charge 0), lowered its solubility. The substitution of a single positively charged residue of T11F (the histidine or one of the arginines), caused a comparable reduction of candidacidal activity. In contrast, the replacement of the only negatively charged residue, aspartic acid, caused a significant increase in candidacidal activity. These findings emphasize the role of the positive net charge of T11F in the interaction with the yeast. Overall, the results are in agreement with the accepted notion that a net positive charge is critical to sustain the activity of antimicrobial peptides [11], [12].

Noteworthy, Fc-peptides did not show hemolytic, cytotoxic or genotoxic activity, as could be expected from peptide fragments potentially occurring in the bloodstream.

Significantly, the selected Fc-peptide N10K, when administered in mouse models of systemic and vaginal candidiasis, displayed a therapeutic activity. In particular, N10K treatment allowed long-term survival of almost half of the mice systemically infected with *C. albicans* in an infection model in which all control animals died within five days post-challenge. Differences in the CFU recovery from the kidneys of treated and control mice strengthened this observation. Equally, N10K treatment proved to significantly accelerate the clearance from the vagina of a high fungal burden in a consolidated experimental model of mouse mucosal candidiasis. N10K greatly accelerated the yeast elimination from mouse vagina in the first 2 to 3 days of infection, a critical period when the infecting yeast cells undergo massive germination to form hyphae and secrete critical virulence enzymes, such as the aspartyl proteinases, thus resulting in aggressive invasion and damage of epithelial cells [17]. Importantly, N10K was also able to eradicate the infection caused by a fluconazole-resistant *C. albicans* strain, a finding of special interest in view of the clinical concern about increasing antifungal resistance [8].

We hypothesize that the therapeutic activity of N10K in the *in vivo* assays could be due to its aggregation ability and, consequently, prolonged life-time. Resistance to protease-induced degradation has been observed for the KP peptide, as previously described [4]. In fact, also in this case the alternation of hydrophobic/hydrophilic residues favours the acquisition of a β-sheet structure and the formation of large aggregates. In addition, the presence of more hydrophobic residues, when compared to the KP peptide, explains the faster aggregation process, *i.e.* days for KP and only hours for N10K.

Electron microscopy observation of N10K-treated *C. albicans* cells may suggest that the peptide does not directly lyse the cells but causes lethal effects seen as cytoplasm retraction and masses of disrupted materials within compartments resembling autophagic structures. Since the cell wall remained intact with many peptide aggregates bound to it, apparently a cytotoxic signal is triggered to induce hydrolytic enzymes that act inside the cell.

These findings may vanish the traditional distinction between adaptive and innate immunity and lead to the hypothesis that, during evolution, the immune system may have adopted an unpredictable range of solutions for host defence against fungal infections. It is now well known that a wide range of proteins contain concealed functional units, that can be released by proteolytic cleavage, with biological activities that cannot be predicted from either the amino acid sequence or the activity of the parent, precursor protein. These “hidden” or “cryptic” peptides were called “cryptides”. They might increase the array of biological roles that can be associated to one given protein and potentially offer new opportunities for peptide-based therapeutics [18], [19], [20], [21]. Cryptides have been divided into three types: peptides detected *in vivo* with functions entirely different from (type I) or related but not necessarily identical to (type II) those of their precursors, and peptides produced *in vitro* by proteolytic digestion of proteins that may or may not exist *in vivo* (type III) [18]. The Fc-peptides described in this paper, which are characterized by antifungal activity, belong to type III, as they were derived from the amino acid sequence analysis of the different classes of Abs by bioinformatic approaches. The demonstration of the presence of Fc-peptides released *in vivo* from antibodies, and of the role played in the antifungal response by these molecules, would be important. Mass spectrometry-based approaches [22] could be extensively used to qualitatively and quantitatively search for these and other Fc-cryptides in human sera from individuals in various clinical conditions. As a matter of fact, Abs could be added to the list of proteins from which cryptides can be derived, so far limited to proteins associated with endocrine signalling, extracellular matrix, complement cascade and milk [20], [21].

Overall, our findings show that synthetic Fc-peptides may, likewise Fab-peptides, exert *in vitro* and *in vivo* fungicidal activities. The demonstration of the presence of these molecules *in vivo* could shed new light on the role that Abs could exert, through their proteolytic fragments, in the antifungal homeostasis. The fungicidal activity of Fc-peptides and their asds allow to hypothesize that Abs may represent a source of new molecules that could serve as prototype drugs exploitable against fungal pathogens.
